# Padlock Probe-Based Generation of DNAzymes for the Colorimetric Detection of Antibiotic Resistance Genes

**DOI:** 10.3390/ijms222413654

**Published:** 2021-12-20

**Authors:** Rick Conzemius, Ariadne Haunold, Ivan Barišić

**Affiliations:** Center for Health and Bioresources, Competence Unit Molecular Diagnostics, AIT Austrian Institute of Technology, Giefinggasse 4, A-1210 Vienna, Austria; rick.conzemius@ait.ac.at (R.C.); ariadne.haunold@gmail.com (A.H.)

**Keywords:** G-quadruplex, DNAzyme, padlock probe, antibiotic resistance, colorimetric detection

## Abstract

The increasing emergence of multidrug- and pan-resistant pathogens requires rapid and cost-efficient diagnostic tools to contain their further spread in healthcare facilities and the environment. The currently established diagnostic technologies are of limited utility for efficient infection control measures because they are either cultivation-based and time-consuming or require sophisticated assays that are expensive. Furthermore, infectious diseases are unfortunately most problematic in countries with low-resource settings in their healthcare systems. In this study, we developed a cost-efficient detection technology that uses G-quadruplex DNAzymes to convert a chromogenic substrate resulting in a color change in the presence of antibiotic resistance genes. The assay is based on padlock probes capable of high-multiplex reactions and targets 27 clinically relevant antibiotic resistance genes associated with sepsis. In addition to an experimental proof-of-principle using synthetic target DNA, the assay was evaluated with multidrug-resistant clinical isolates.

## 1. Introduction

The emerging antibiotic resistance crisis represents a serious healthcare problem and was declared by the WHO as one of the biggest threats to global health, food security, and development. For the implementation of efficient disease control measures, cost-effective and fast diagnostic systems are essential tools to identify local outbreaks and to contain the further spread of multi- (MDR) and pandrug-resistant (XDR) pathogens into the community. The current gold standard in clinical microbiology for the characterization of antibiotic resistance is based on cultivation-based techniques that are very cost-effective but require several days until the results become available. In contrast, DNA-based technologies such as real-time PCR are fast (<4 h) but still too expensive for mass screenings in low-resource settings. In particular, the application of fluorescent labels and light cyclers are major cost-factors in such assays. Thus, novel technologies that are significantly more cost-effective and able to detect hundreds of antibiotic resistance genes in parallel have still to be developed.

**Figure 1:**
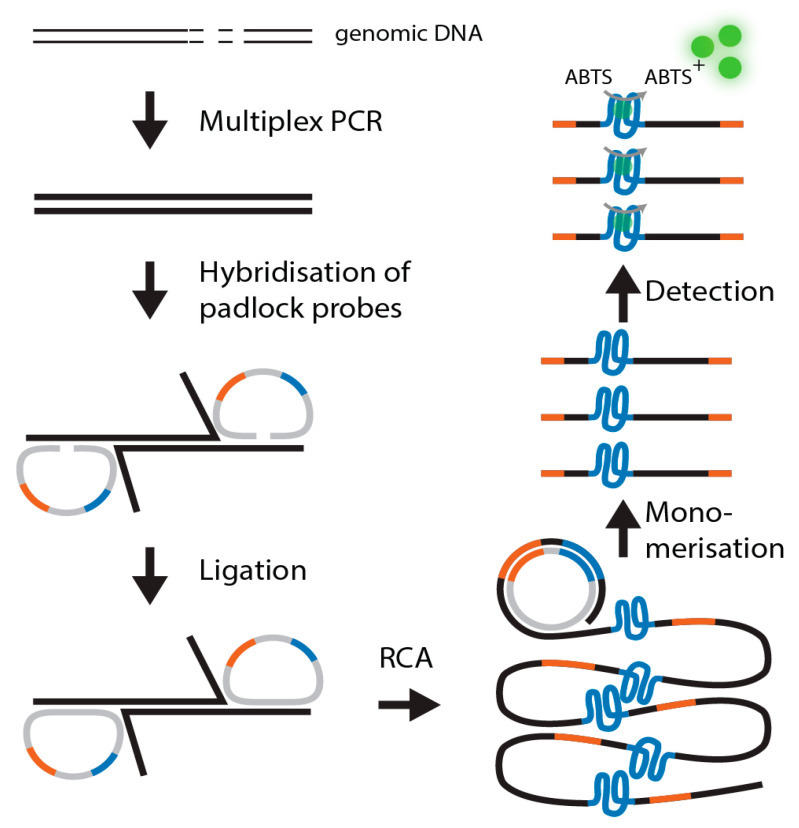
G-quadruplex padlock probe protocol. First, genomic DNA is amplified by a 45-plex PCR targeting antibiotic resistance genes. Then, padlock probes hybridize to the target. Next, the probes are ligated and circularized. Circularized probes are amplified in an RCA. The probes are then monomerized by enzymatic digestion, and finally, the active DNAzyme is detected in a colorimetric reaction.

Padlock probes are long, single-stranded oligonucleotides with two terminal recognition regions complementary to a target, a primer attachment site, and usually a DNA barcode sequence (Figure 1) [1]. Upon hybridization of the two ends to a target, the probe circularizes without a gap between the 3′ and 5′ ends. The probe is then ligated by a highly specific ligase.

This ligated circular probe can then be specifically replicated using rolling circle amplification (RCA) to a very high copy number. The resulting RCA products are usually detected via real-time amplification or microarrays [2,3]. High specificity is guaranteed due to the higher specificity of the ligase (compared to a polymerase) and the double recognition by two target recognition arms allowing to distinguish single nucleotide polymorphisms (SNPs) [4]. Multiplexing can be done to a very high degree of up to 10,000 probes [5,6]. However, the degree of multiplexing is limited by the downstream analysis techniques. While microarrays allow analyzing 10,000 targets in parallel, this is not possible using quantitative PCR. The padlock probe technique was already used for the detection of pathogens and their antibiotic resistance genes [7,8].

**Figure 2:**
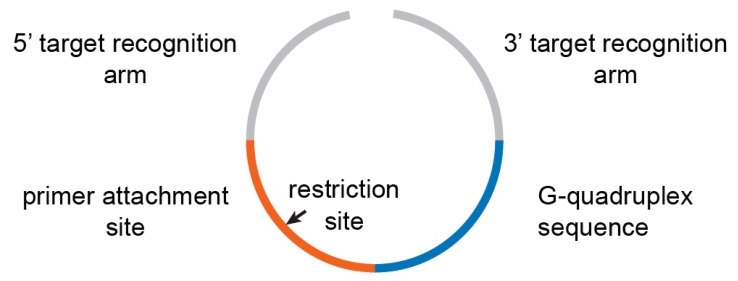
G-quadruplex padlock probes contain two terminal target recognition arms to the target of interest, a primer binding site with a restriction site, and a G-quadruplex sequence.

Here, we describe a novel detection system independent of a costly downstream analysis platform. We integrated a complementary G-quadruplex-hemin DNAzyme sequence into the padlock probe. This sequence is amplified during an RCA and finally monomerized using a restriction site integrated into the primer attachment site. The sequence is then detected using the DNAzyme-catalyzed peroxidation of the 2,2′-azino-bis(3-ethylbenzothiozoline-6-sulfonic acid) diammonium salt (ABTS2-), which is visible by the naked eye. The scheme of the assay is shown in Figure 2. Although this assay relies on amplification techniques, the assay is considered label-free since no fluorescent, chemiluminescent, radioactive, or electrochemical readout methods were used.

## 2. Results

### 2.1. Design of the G-Quadruplex Padlock Probes

Padlock probes targeting 45 clinically important antibiotic resistance genes associated with pathogens causing bloodstream infections and sepsis were designed. After the specificity evaluation, 18 probes were removed from the panel due to being unspecific (data not shown), resulting in a 27-plex panel. Each padlock probe is flanked at the 3’ and 5’ ends by two arms complementary to their corresponding antibiotic resistance gene. Upon successful hybridization of both arms to the genetic target, the molecule is circularized and can further be amplified using RCA. Additionally, each padlock probe encodes a G-quadruplex sequence (A4G4) and contains a primer-annealing site for the initiation of the RCA. The primer annealing sequence comprises also an AvaI restriction site. The specificity of the probes was assured using PRIMEval [9].

### 2.2. Optimization of PCR, RCA, Restriction, and Detection Buffer

**Figure 3:**
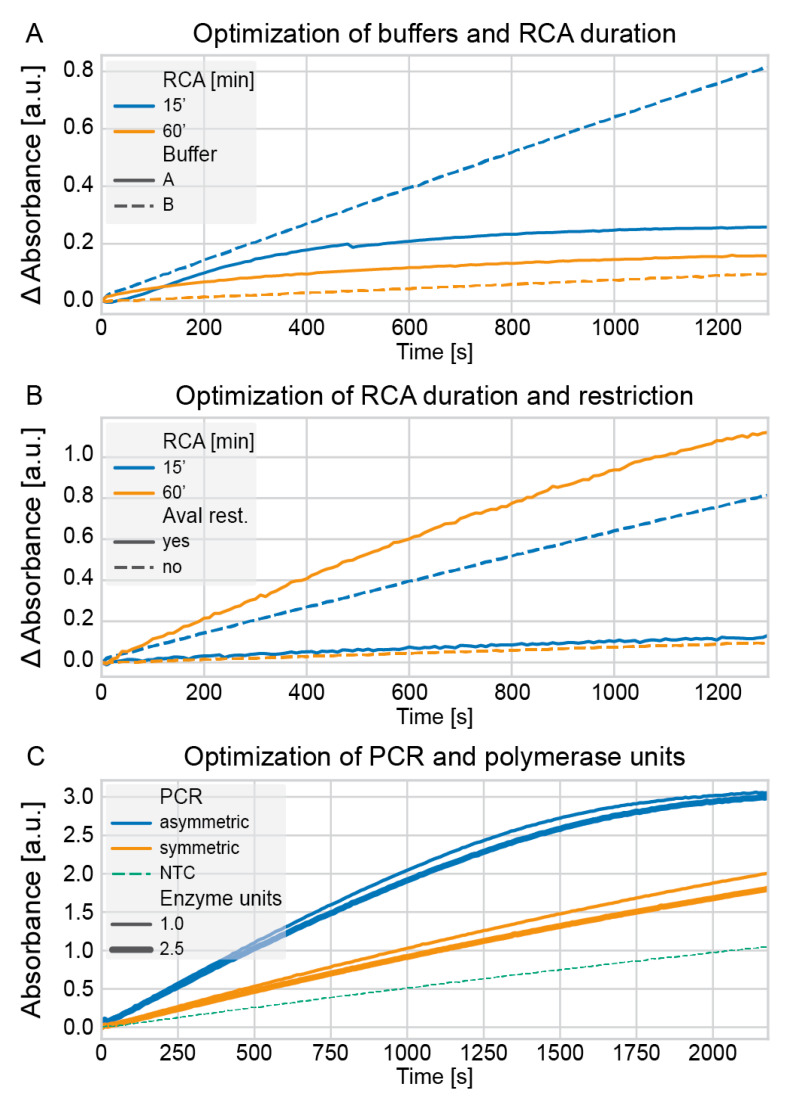
Optimization of the PCR, RCA, restriction, and detection buffer. The amplification and detection of the *CTX-M-1* gene were used as a representative example. (**A**) The RCA was carried out for 15 or 60 min, and the detection was done in buffers of different compositions and pH. (**B**) The RCA was carried out for 15 or 60 min, and the RCA products were restricted by the addition of the AvaI restriction enzyme. (**C**) A symmetric PCR was compared to an asymmetric PCR, and the number of polymerase units was evaluated. In (**A**,**B**), the absorbance is normalized to the corresponding negative controls. In contrast, in (**C**), the absolute absorbance is given, and the signal of the negative control is shown on the graph.

We optimized several conditions to transfer our previously published multiplex padlock protocol intended for a microarray-based detection platform [10] to the new platform using colorimetric detection. First, we evaluated buffers for the incubation of the RCA products with hemin to allow the formation of active G-quadruplex DNAzymes with peroxidase activity. Two buffers which differ by their addition of 0.05% Triton X-100 and 1% dimethyl sulfoxide (DMSO), and their pH, were compared. In addition, we investigated whether the length of the RCA products has an impact on the formation and detectability of DNAzymes. Therefore, RCA incubation times of 15 min and 60 min were compared. The results from multiple experiments indicated that the buffer with the lower pH and without Triton X-100 and DMSO (buffer B) produces a higher signal to noise difference if the RCA takes place for only 15 min. If the RCA is incubated for 60 min, barely a signal difference is detectable, independent of the buffer used (Figure 3A). We hypothesized that the long RCA molecules are inhibiting the detection by producing tertiary structures which mask the DNAzymes.

To undermine this hypothesis that long RCA products are inhibiting, we cleaved the RCA products to shorter products using a restriction site in the primer sequence of the padlock probe and enzyme AvaI. If the RCA products were monomerized to individual molecules complementary to the padlock probe containing a single DNAzyme, the activity was higher if the RCA was incubated for 60 min, i.e., if more DNAzymes were generated. If the products were not enzymatically cleaved, the detection was more efficient if the RCA was carried out for only 15 min (Figure 3B). This confirms that more generated DNAzymes lead to an increase in signal, but only if the generated molecules are monomers or small polymers instead of very long RCA products. The general RCA reaction and the follow-up restriction step was verified on agarose gels (Figure S2).

Last, we evaluated the optimal number of polymerase units to use in the preamplification of the targets from clinical isolates and whether asymmetric PCR is advantageous over symmetric PCR. We hypothesized that the generation of single-stranded target molecules should allow a more efficient hybridization of the padlock probe to the target since no strand invasion into the double-stranded PCR product has to take place. Indeed, as can be seen in Figure 3C, asymmetric PCR generated higher signals than symmetric PCR. The impact of different concentrations of the polymerase was negligible. In addition, all signals were significantly above the negative control, which consists of a no-template PCR product. However, the buffer composition, dNTPs, proteins, and primers in the PCR product contribute to the increase in measured absorbance [11,12,13,14,15].

### 2.3. Specificity Evaluation of Padlock Probes Targeting Antibiotic Resistance Genes with Synthetic DNA

**Figure 4:**
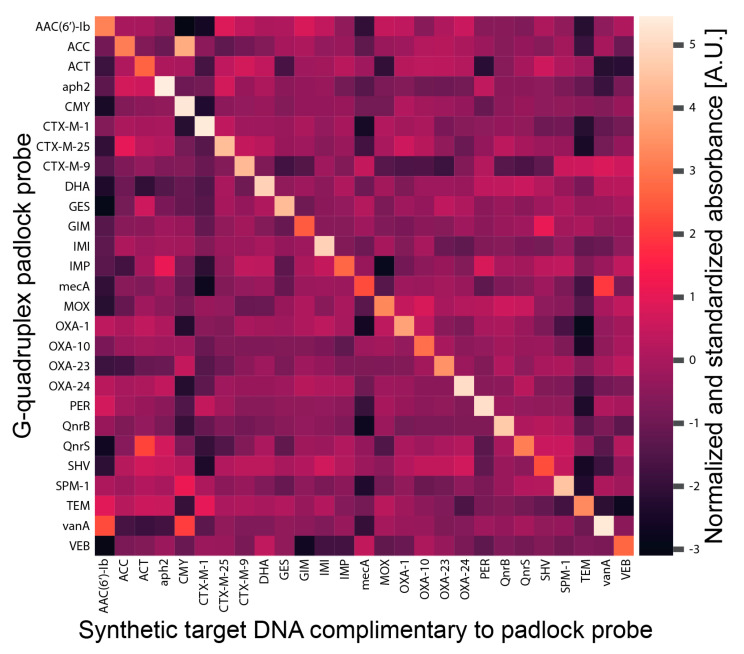
Specificity evaluation of 27 padlock probes targeting antibiotic resistance genes with synthetic DNA. Each target sequence was tested against all 27 padlock probes. The padlock probes were ligated, amplified, and finally, the conversion of the ABTS chromophore by the G-quadruplex DNAzymes was measured.

For the functionality evaluation of every padlock probe, we used short, complementary, single-stranded target oligonucleotides for each padlock probe. Every single-stranded target sequence was incubated with every padlock probe. This led to the ligation and circularization of the padlock probe if the intended target was present. In contrast, the padlock probe should remain linear in the absence of target DNA. The ligated padlock probes were then amplified using RCA. At this step, the complementary DNAzyme sequence was transcribed and could fold into the DNAzyme (G-quadruplex) configuration. The colorimetric reaction was then started by the addition of hemin, ABTS, and hydrogen peroxide (H_2_O_2_). Although the probes generated high background signals during the specificity evaluation, we showed that each probe was specific to its intended target (Figure 4). Because G-rich oligonucleotides can form inter- and intramolecular G-quadruplexes causing these strong background signals, each probe requires an individual normalization. We concluded that the generation of signals is highly dependent on the target present in the reaction and each probe, if specific, requires its own normalization as determined during the specificity evaluations [15,16].

### 2.4. Evaluation of Padlock Probes Targeting Antibiotic Resistance Genes with Genomic DNA

We evaluated all 27 padlock probes with DNA extracted from clinical isolates of *Enterococcus faecium*, *Klebsiella pneumoniae*, *Acinetobacter baumannii*, and *Enterobacter cloacae*. For this purpose, all padlock probes were incubated with the extracted DNA (amplified in an upstream 45-plex PCR) and with H_2_O as a negative control. The padlock probes were then ligated and amplified using RCA. Finally, the conversion of the ABTS chromophore by the G-quadruplex DNAzymes was measured. Signals from the samples with H_2_O only were subtracted from their counterparts with genomic DNA, leading to a normalization specific to each padlock probe, but not to the target as stated above.

**Figure 5:**
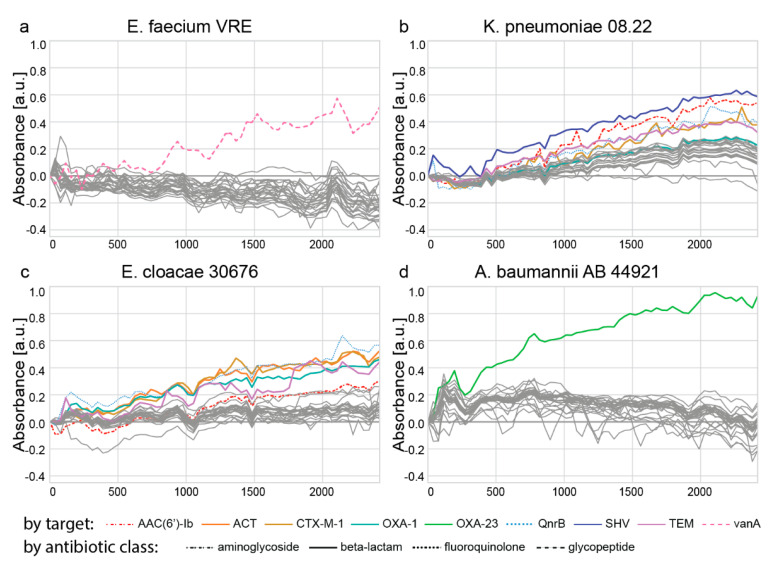
Evaluation of 27 padlock probes targeting antibiotic resistance genes. The genomic DNA of four clinical isolates of the species (**a**) *E. faecium*, (**b**) *K. pneumoniae*, (**c**) *E. cloacae*, and (**d**) *A. baumannii* was used. Each clinical isolate was incubated with all 27 padlock probes (in single reactions); in parallel, a negative control (H_2_O) was incubated with all 27 padlock probes. The padlock probes were ligated, amplified, and finally, the conversion of the ABTS chromophore by the G-quadruplex DNAzymes was measured. Intended targets per clinical isolate are colored and indicated below the figure; the class of the target antibiotic compound is indicated by the line type.

The evaluation with clinical isolates is shown in Figure 5a–d. For *E. faecium* VRE and *A. baumannii* AB44921, the highest signals are produced by the intended targets, which are *vanA* or *OXA-23*, respectively. With *K. pneumoniae* 08.22, 5 of the 6 highest signals belong to the intended signals (*AAC(6′)-Ib, CTX-M-1, QnrB, SHV, TEM*), but the probe corresponding to *OXA-1* was not differentiable from the background signal. The *E. cloacae* 30676 isolate produced the 5 highest signals for all intended targets (*ACT, CTX-M-1, OXA-1*, *QnrB, TEM*), but the *AAC(6′)-Ib* was not detected significantly above the background.

## 3. Discussion

In summary, we have demonstrated the successful proof-of-principle that G-quadruplex-based DNAzymes can be used in combination with padlock probes making a colorimetric readout in the presence of a target gene possible. We designed the padlock probes to have a length of 80 nt which is optimal regarding the amplification efficiencies of the polymerase during the RCA [17]. We optimized the reaction conditions using different PCR setups, durations of the RCAs, restriction digestion of the RCA products, and different detection buffers [11,12,13,14,15,16]. However, future optimizations that have a strong impact on the specificity of the reaction must include the padlock probe design and the RCA. In an analogous study using a microarray platform, we have observed non-specific RCA products even in the presence of only a single synthetic target gene in the assay [8]. This phenomenon also observed in our present study indicates that in high-multiplex reactions the specificity of the DNA-based detection and amplification reactions must be intensively optimized. Further improvements of the method could also focus on the reduction of the effect that residual NTPs have on the G-quadruplex reactivity [15]. Additionally, the DNAzyme activity could be enhanced by flanking the G-quadruplex sequence with d(CCC), which improves pH tolerance and hemin binding to the DNAzyme [18]. Alternatively, the addition of polyamines such as spermidine, spermine, or putrescine, can stabilize G-quadruplex/hemin complexes [19]. In the future, multiple padlock probes specific to a single target should be designed to increase the specificity and pooled for better signal-to-noise ratios improving the sensitivity. Alternative steps to further optimize this detection technology require large-scale screenings in different setups to generate enough data for normalization procedures that improve the signal-to-noise ratios.

In conclusion, our detection principle is most suited in low-resource settings when the parallel detection of several genes has to be performed. Using a colorimetric readout and isothermal enzymatic reactions for the ligation and DNA amplification allows the development of detection assays that are independent of sophisticated and expensive instruments. Additionally, using DNAzymes that are encoded in the padlock probes reduces the complexity of the reaction because the application of additional reporter enzymes (e.g., HRP), washing and incubation steps can be avoided. In comparison to the qPCR or the loop-mediated isothermal amplification (LAMP), which is also well-suited for low-resource settings, our padlock probe-based method can be multiplexed to a significantly higher degree and also allows the precise detection of single nucleotide polymorphisms [2,8]. The high-multiplexing capability is of special importance where a high number of potentially present genes has to be screened such as in clinical microbiology. We successfully applied these features to detect a wide range of genetically diverse antibiotic resistance genes.

## 4. Materials and Methods

Chemicals, enzymes, and buffers. The ampligase thermostable ligase was obtained from Lucigen (Middleton, WI, USA), dNTPs from Invitrogen (Thermo Fisher Scientific, Waltham, MA, USA), phi29 DNA polymerase, AvaI restriction enzyme, and bovine serum albumin (BSA) from New England Biolabs (Ipswich, MA, USA), hemin from Merck KGaA (Darmstadt, Germany), 2,2′-azino-bis(3-ethylbenzothiazoline-6-sulfonic acid) (ABTS) tablets and hydrogen peroxide solution (H_2_O_2_) from Sigma–Aldrich (St. Louis, MO, USA), and Hot MolTaq 16S/18S polymerase from Molzym (Bremen, Germany). The EnSpire multimode plate reader was from PerkinElmer (Waltham, MA, USA). The Ion Torrent sequencing chemistries were obtained from Thermo Fisher Scientific (Waltham, MA, USA). HEPES buffer A consisted of 25 mM of HEPES, 20 mM of KCl, 200 mM of NaCl, 0.05% Triton X-100, and 1% DMSO (pH 7.4 @ 25 °C). HEPES buffer B consisted of 25 mM of HEPES, 20 mM of KCl, and 200 mM of NaCl (pH 5.5 @ 25 °C).

Oligonucleotides. All oligonucleotides (Supplementary Tables S1–S4) were obtained from Integrated DNA Technologies (Coralville, IA, USA). Each padlock probe consists of a sequence 1, insert 1, filler, insert 2, and sequence 2 as indicated in Supplementary Table S1; the insert 1 (primer attachment site) corresponds to ACTACTTACCTCGGGC, and the insert 2 (G-quadruplex sequence) corresponds to TTTTCCCCTTTTCCCCTTTT-CCCCTTTTCCCC.

Design of the G-quadruplex padlock probes. The padlock probes were designed using a publicly available Python script. The script takes the sequences from the products of the 45-plex primer set (Supplementary Table S2) as input, then complementary sequences are designed starting at either strand with an offset to the 5’ end of 2–25 bp (Tm = 62 °C). Thermodynamic data were calculated using SantaLucia’s model with salt correction. The complementary G-quadruplex sequences and a primer attachment sequence were inserted into the padlock probe. If the target length was shorter than 80 nt, a random filler sequence was inserted. The probes are listed in Supplementary Table S1. Initially, 45 probes were designed to cover the full 45-plex PCR assay, but due to their false-positive and interfering signals, 17 probes were removed from the assay, resulting in a functional 27-plex detection assay.

Bacterial culture and DNA extraction. A volume of 10 µL of bacterial suspension from clinical isolates (*Enterococcus faecium* VRE, *Klebsiella pneumoniae* 08.22, *Enterobacter cloacae* 30676, *Acinetobacter baumannii* 44921) was inoculated in 5 mL of TSB. The incubation took place at 37 °C in a 15-mL conical tube on an orbital shaker set to 300 rpm for 20 h. DNA of all strains was extracted and purified using the QIAamp DNA Mini Kit (QIAGEN, Hilden, Germany). The first steps of the manufacturer’s protocol comprising cell lysis and disruption: A total of 2 mL of cell suspension was taken from liquid culture. The total volume was centrifuged for 5 min at 5000× *g*, then the supernatant was discarded, and the cell pellet was resuspended in 200 µL of TSB. The resuspended pellet was then added to a 2-mL vial with a screw cap containing sterile acid-washed glass beads with a diameter of 150–212 µm (Sigma–Aldrich, St. Louis, MO, USA) filled to the 100-µL mark. The suspension was subjected to 2 runs at 6500 rpm for 30 s in a MagNA Lyser instrument (Roche, Basel, Switzerland), separated by a 5 min incubation at room temperature. The vial was centrifuged for 3 min at 16,000× *g*, and 180 µL of the supernatant was transferred to a clean 1.5-mL Eppendorf tube. The protocol was continued with the addition of buffer AL and proteinase K according to the manufacturer’s instructions. The DNA concentration was measured on a NanoDrop 2000c spectrophotometer (Thermo Fisher Scientific, Waltham, MA, USA).

Sequencing. Whole-genome sequencing was performed on the Ion Torrent PGM platform (Thermo Fisher Scientific, Waltham, MA, USA) using reads with a length of 400 nt. Library preparation was done using the Ion Xpress Plus Fragment Library kit, 100 ng of genomic DNA input, and Ion Xpress Barcode Adapters, following the manufacturer’s instructions. The sheared DNA was size-selected on E-Gel SizeSelect II agarose gels (Thermo Fisher Scientific, Waltham, MA, USA). The total volume was recovered. Quantification was done using the Ion Library TaqMan quantitation kit following the manufacturer’s instructions on a LightCycler 480 System (Roche, Basel, Switzerland). The library was quantified and qualified on a 5300 Fragment Analyzer System (Agilent Technologies, Santa Clara, CA, USA) using the HS Small Fragment Kit (Agilent Technologies, Santa Clara, CA, USA). All samples were diluted to 100 pM, and 5 µL of each sample were pooled together. The sample for sequencing was prepared using the Ion PGM Hi-Q View OT2 kit following the manufacturer’s instructions. Clonal amplification and enrichment were carried out with the Ion OneTouch 2 instrument. Template-positive ion sphere particles (ISPs) were enriched on the Ion OneTouch ES instrument. The quality of unenriched, template-positive ISPs was assessed using the Ion Sphere Quality Control kit on the Qubit 2.0 fluorometer (Thermo Fisher Scientific, Waltham, MA, USA) following the manufacturer’s instructions. The sequencing was performed using the Ion PGM Hi-Q View Sequencing kit following the manufacturer’s instructions, except that control ISPs were not added to the sample. The chip loading was done per the manufacturer’s instructions on an Ion 318 chip. The reads were analyzed using FastQC. If necessary, additional trimming and filtering were done using PRINSEQ [20]. The reads were assembled into contigs and scaffolds using SPAdes [21] 3.11.1 (parameters: iontorrent, k: [21], repeat resolution enabled, mismatch careful mode turned off, MismatchCorrector skipped, coverage cutoff turned off). The quality of the assemblies was assessed using QUAST 4.6.3 [22]. The scaffolds were uploaded to the European Nucleotide Archive (ENA). The antibiotic resistances were identified using ResFinder 3.2 [23] (parameters: acquired antimicrobial resistance genes: checked, threshold for %ID = 60%, minimum length = 60%). The specificity of primers and padlock probes was checked using PRIMEval [9].

Optimization of PCR, RCA, restriction, and detection buffer. A PCR (50 µL) comprised 1 × PCR buffer (3.0 mM of MgCl2), 200 µM of each dNTP, 0.02 or 0.05 units/µL Hot MolTaq 16S/18S polymerase (as indicated), and two corresponding primers (Supplementary Table S2) at 400 nM each. If asymmetric PCR was carried out, the forward primer was used at a final concentration of 1200 nM and the reverse primer at 40 nM. The target DNA was added to a final concentration of 0.4 ng/µL. The thermal cycling was performed as follows: 94 °C for 5 min; 40 cycles at 94 °C for 30 s, 49 °C for 30 s, 72 °C for 30 s; and a final elongation at 72 °C for 7 min. The ligation reaction (45 µL) comprised 1 × ampligase reaction buffer, 50 nM of the padlock probe targeting the gene of interest, 0.2 µg/µL of BSA, 0.20 units/µL of ampligase thermostable DNA ligase, and 10 µL of PCR products. The reaction was incubated for 5 min at 95 °C and 15 min at 55 °C. The rolling circle amplification (RCA) (40 µL) comprised 30 µL of the ligation products, 1 × phi29 DNA polymerase reaction buffer, 0.2 µg/µL BSA, 125 µM of each dNTP, 100 nM of padlock probe primer (Supplementary Table S4), and 0.17 units/µL phi29 DNA polymerase. The RCA was incubated for either 15 min or 60 min at 37 °C, then 2 min at 65 °C. If an enzymatic restriction was done, 10 µL of the restriction mix was added to 40 µL of the RCA product. The restriction mix comprised 1 × phi29 DNA polymerase reaction buffer, 0.2 µg/µL BSA, 1 µM of the reverse complementary padlock probe primer (Supplementary Table S4), and 2 units/µL AvaI restriction enzyme. The restriction reaction was incubated for 30 min at 37 °C and 10 min at 80 °C. The detection of the formed G-quadruplex DNAzymes was done by mixing 5 µL of the (digested) RCA products with 70 µL of the detection mix, consisting of 1 × HEPES buffer A or 1 × HEPES buffer B and 10 µM hemin. The colorimetric reaction was started by the addition of 5 mM of ABTS and 1 mM of H_2_O_2_. All indicated concentrations are final concentrations in the detection volume of 100 µL. The absorbance at 421 nm was measured for up to 40 min on an EnSpire multimode plate reader.

Specificity evaluation of padlock probes targeting antibiotic resistances with synthetic DNA. The 27 padlock probes (Supplementary Table S1) and 3 controls without probes (H_2_O) were put into one half of a 96-well plate (final concentration in the reaction: 50 nM). Then a master mix containing 1 × ampligase reaction buffer, 0.34 units/µL of ampligase thermostable DNA ligase, and 500 nM of synthetic DNA target (Supplementary Table S3) was added in a final volume of 5 µL. For the ligation, the plate was incubated for 5 min at 95 °C and 15 min at 55 °C. Then, 5 µL of an RCA reaction mix was added to each well. This reaction mix consisted of 1 × phi29 DNA polymerase reaction buffer, 0.2 µg/µL of BSA, 125 µM of each dNTP, 100 nM of padlock probe primer (Supplementary Table S4), and 0.17 units/µL of phi29 DNA polymerase. All indicated concentrations are final concentrations in the reaction volume of 10 µL. The RCA was incubated for 15 min at 37 °C and 10 min at 80 °C; then, 5 µL of the RCA products were transferred to a flat-bottom plate. The formed G-quadruplex DNAzymes were detected by the addition of the detection mix, consisting of 1 × HEPES buffer B and 10 µM of hemin. The reaction was started by the addition of 5 mM of ABTS and 1 mM of H_2_O_2_. All indicated concentrations are final concentrations in the detection volume of 100 µL. The absorbance at 421 nm was measured for 40 min on an EnSpire multimode plate reader. The data were normalized to the first time point and to the “no probe control”. Individual graphs were generated using the seaborn library. All end points were summarized in a heatmap generated by the seaborn library after normalization using StandardScaler of scikit-learn. In Figure S1, the data are also normalized using Standard Scaler as represented in the heatmap. 

Evaluation of padlock probes targeting antibiotic resistances with genomic DNA. The 45-plex PCR (55 µL) comprised 1 × PCR buffer (3.0 mM of MgCl2), 200 µM of each dNTP, 0.05 units/µL Hot MolTaq 16S/18S polymerase, and primers targeting antibiotic resistance genes (Supplementary Table S2) at 111 nM each (10-µM final concentration). The target DNA was added to a final concentration of 0.4 ng/µL. The thermal cycling was performed as follows: 94 °C for 5 min; 30 cycles at 94 °C for 30 s, 49 °C for 30 s, 72 °C for 30 s; and a final elongation at 72 °C for 7 min. The 27 padlock probes (Supplementary Table S1) and 3 controls without probes (H_2_O) were put twice into a 96-well plate (final concentration in the reaction: 50 nM), then a master mix containing 1 × ampligase reaction buffer, 0.34 units/µL of ampligase thermostable DNA ligase, and 1 µL of PCR product or H_2_O per reaction was added. The final reaction volume was 5 µL. The plate was incubated for 5 min at 95 °C and 15 min at 55 °C. Then, 5 µL of an RCA mix was added to each well. This reaction mix consisted of 1 × phi29 DNA polymerase reaction buffer, 0.2 µg/µL of BSA, 125 µM of each dNTP, 100 nM of padlock probe primer (Supplementary Table S4), and 0.17 units/µL phi29 DNA polymerase. All indicated concentrations are final concentrations in the reaction volume of 10 µL. The RCA was incubated for 15 min at 37 °C and 10 min at 80 °C; then, 5 µL of the RCA products was transferred to a flat-bottom plate. The formed G-quadruplex DNAzymes were detected by the addition of the detection mix, consisting of 1 × HEPES buffer B and 10 µM of hemin. The reaction was started by the addition of 5 mM of ABTS and 1 mM of H_2_O_2_. All indicated concentrations are final concentrations in the detection volume of 100 µL. The absorbance at 421 nm was measured for 40 min on an EnSpire multimode plate reader. For the data analysis, the negative controls were subtracted from the positive controls, then the first time point was subtracted from all other time points, and finally, the ‘no probe control’ was subtracted from all values at every time point. Individual graphs were generated using the seaborn library.

## Data Availability

The assemblies of the sequences of the clinical strains are accessible using following accessions: CABFJB010000000 (*E. faecium* VRE), CABFJE010000000 (*K. pneumoniae* 08.22), GCA_900465105 (*E. cloacae* 30676), and CABFJO010000000 (*A. baumannii* 44921). The Python script for the design of the padlock probes is available under https://github.com/rczms/g4_pp (access on 27 July 2020).

## Supplementary Materials

The following are available online at https://www.mdpi.com/article/10.3390/ijms222413654/s1.
