# Supplementary material for: Padlock Probe-Based Generation of DNAzymes for the Colorimetric Detection of Antibiotic Resistance Genes
Source: Int J Mol Sci. 2021 Dec 20;22(24):13654. doi: 10.3390/ijms222413654 (PMC8715668; doi:10.3390/ijms222413654)
Supplement: Supplementary file 1 [file ijms-22-13654-s001.zip › ijms-1498463 -supp update.pdf]

Supplementary information

Padlock probe-based generation of DNAzymes for the colorimetric detection of antibiotic resistance genes

Rick Conzemius, Ariadne Haunold, and Ivan Barišić

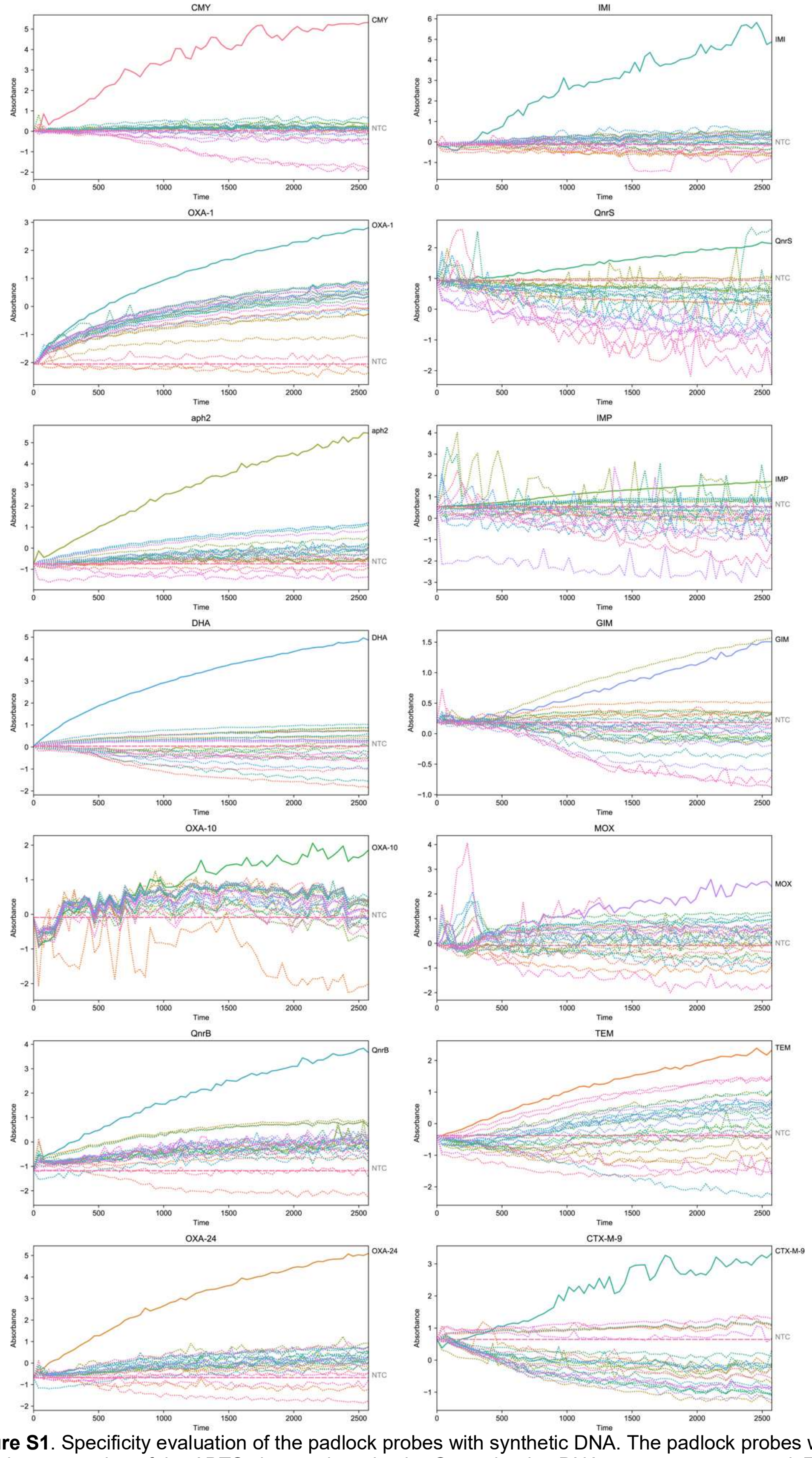

Supplementary Figure S1. Specificity evaluation of the padlock probes with synthetic DNA. The padlock probes were ligated, amplified, and finally, the conversion of the ABTS chromophore by the G-quadruplex DNAzymes was measured. The data was normalized to the first time point and the 'no probe control'. The curve was smoothened using a rolling average (window: 5 data points). Continued on the next page.

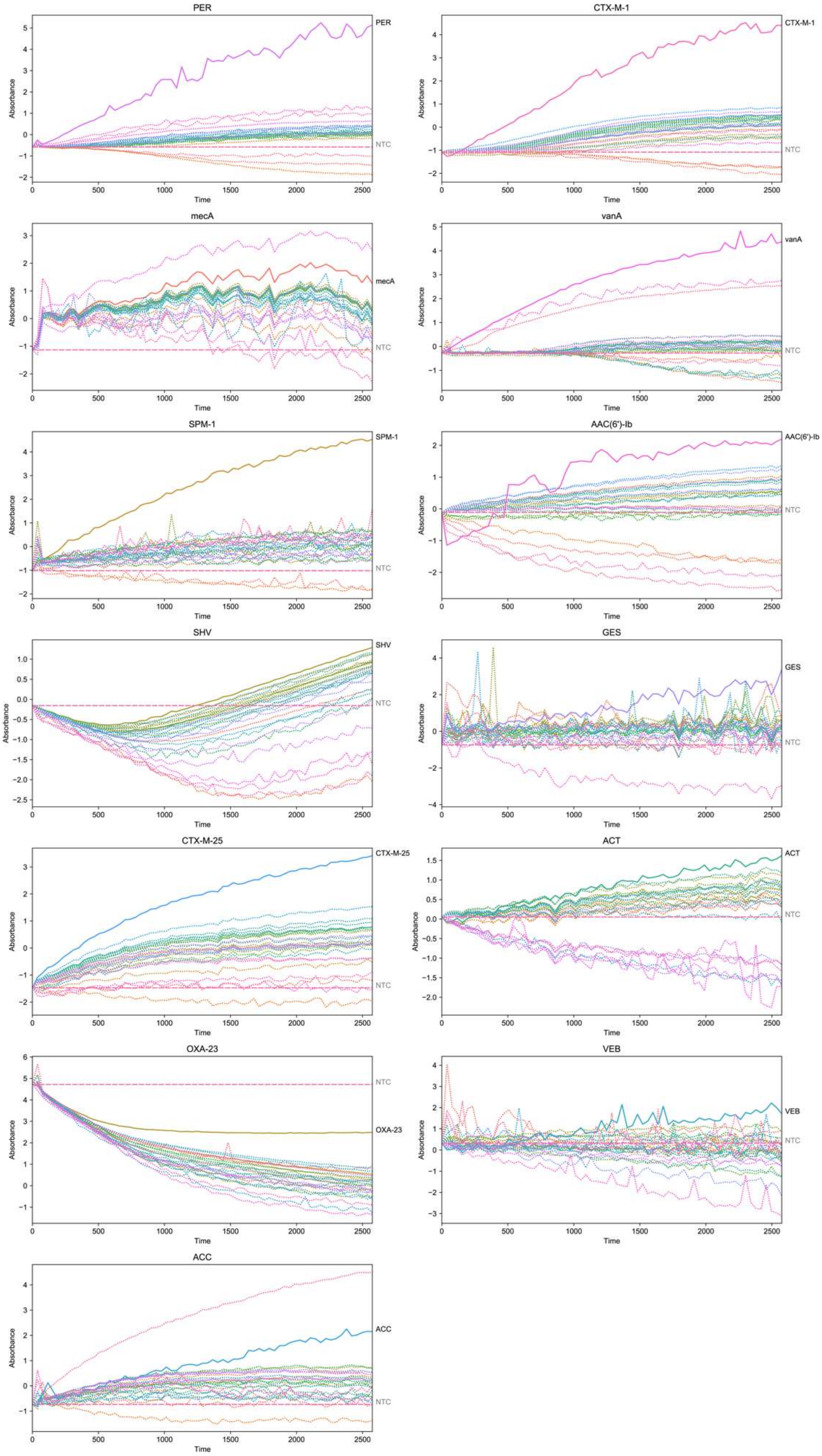

Supplementary Figure S1 (cont'd). Specificity evaluation of the padlock probes with synthetic DNA. The padlock probes were ligated, amplified, and finally, the conversion of the ABTS chromophore by the G-quadruplex DNAzymes was measured. The data was normalized to the first time point and the 'no probe control'. The curve was smoothened using a rolling average (window: 5 data points).

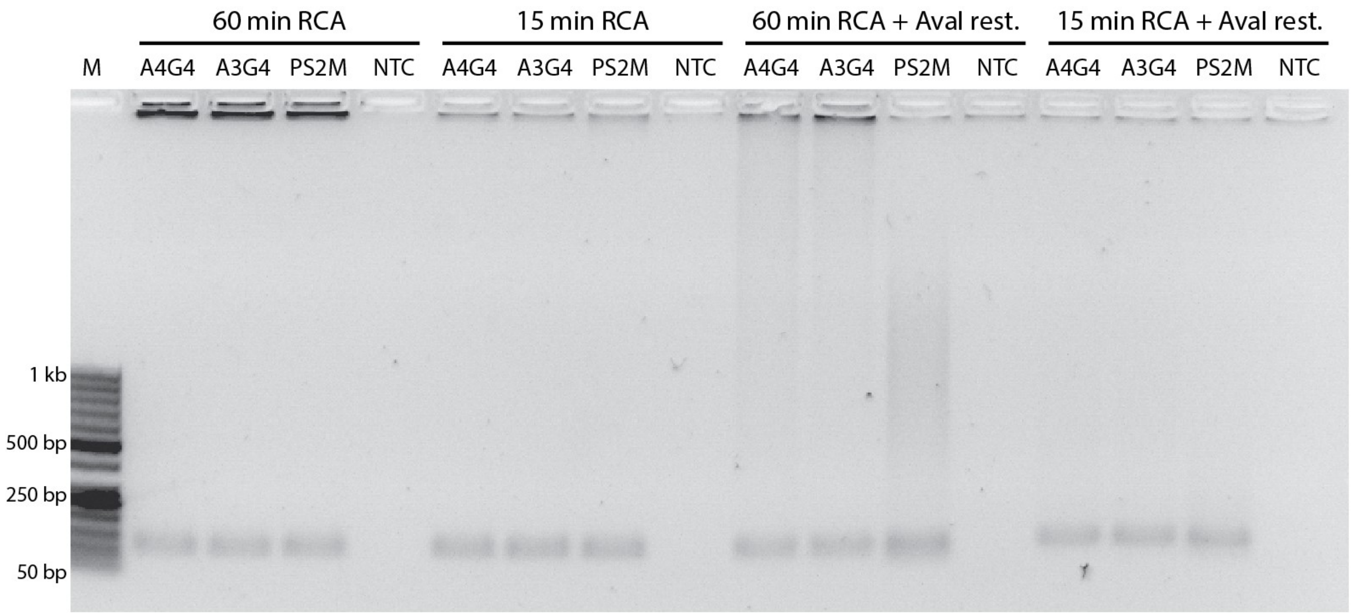

Supplementary Figure S2. Verification of the RCA and restriction digestion by AvaI. The agarose gel shows the successful amplification of the CTX-M-1 gene after A) a RCA of 60 min, B) a RCA of 15 min, C) a RCA of 60 min followed by AvaI restriction, and D) a RCA of 15 min followed by AvaI restriction. The labels above the gel pockets represent different G-quadruplex DNAzyme sequences which were evaluated. The final design used in this publication is A4G4.

Supplementary Table S1. G-quadruplex padlock probes. Each padlock probe consists of the sequence 1, insert 1, filler, insert 2 and sequence 2; all sequences are in the 5'-3' order. The insert 1 sequence corresponds to ACTACTTACCTCGGGC, and the insert 2 sequence corresponds to TTTTCCCCTTTTCCCCTTTTCCCCTTTTCCCC.

Name	Sequence 1	Filler	Sequence 2	Tm 1 [°C]	Tm 2 [°C]	Length [nt]	5' offset
AAC(6')-Ib	CGCTTCCAAGAGCAACG		TCCCACCATCCGTCCC	61	62	81	11
ACC	GGGGATCGTGATGCTGG		CGTTTGTTCCTGCTAAGAAGAT	61	61	87	20
ACT	CGCCAACGCCAGCA		TGGCACAACGCGTCTTTA	61	62	80	13
aph2	GCATAAGGCGCCGGAA		GGCAGGAAGGTATTAAAATACTATCA	62	62	90	18
CMY	GCGCTTCCCGCCG	A	AGCAAAGTGGCATTGGCA	61	63	80	5
CTX-M-1	TGCGCATACAGCGGC	C	CTGTACGTCCGCCGTT	62	61	80	9
CTX-M-25	CGCCGCTACCGCC		CGTTTCACTTTGCTTAAGCAC	60	61	82	12
CTX-M-9	GCCGCTGGTTCTGGT	TTG	CCGCAGGGTCGTGC	60	61	80	21
DHA	GCGGTTTCTGTGGCGA		TCACAGGTGTGCTGGGT	62	62	81	8
GES	CTACGAGCGGGTTTTCCT		AACGGGAGACGCGACA	61	62	82	17
GIM	GGTAGTTCTGGATAATAATCAAGCC		GGTTAGTTGATTCGAATGGGTT	62	61	95	19
IMI	AATCGTTCATTTGCTTTGTACGA		GCCTTTGAAAGAACTACATAATGGA	62	62	96	11
IMP	ATAGATCGAGAATTAAGCCACTCT		TTCAGATGCATACGTGGGG	62	62	91	5
mecA	AGTTGAACCTGGTGAAGTTGT		CCCAATCATTGCTGTTAATATTTTTTG	62	62	96	15
MOX	GGCGAGTTATGCCTATGGC		GCCGAAGCAGGCCAT	63	61	82	19
OXA-1	AGAACCTTACAAAACGGATGGT		GTGCAGGATTCACAGCAAAT	63	61	90	17
OXA-10	TGCGACACCAGGATTTGAC		CCCACCCAACCCACCA	63	63	83	20
OXA-23	ATATGTGCCAGCCTCTACATTT		AAGCCGCGCAAATACAGA	62	62	88	22
OXA-24	ACTCCACAGGTAGGTTGGT		GGATGGGGAATGGGTGTT	62	61	85	7
PER	TGCAGCAGTTGCTGCA		GTGTGCGAGACCGAGTAT	62	61	82	16
qnrB	AACTCGCCGCCGGA		CGCCAGTCGAAAGTCGAA	62	62	80	20
qnrS	CGGGAAAAGTTGGCACCT		ACTTGATGGGCAAAGTTTGTT	62	61	87	12
SHV	GGGTGCGCGCGG	CACTGA	GGAGCTGGCGAGCG	61	61	80	18
SPM-1	CCGCGCCCATCTTCTT		TCGCTCGACCACGTCT	61	62	80	4
TEM	GTGATAACACTGCGGCCA		AGTGCTGCCATAACCATGA	62	61	85	13
vanA	GGTCTAGCCCGTGTGGA		AGCGCTCGGCTGTAGA	62	62	81	20
VEB	CCACGTTTTAGGCAAAAGGT		GGGAATTCCTCTTTAATCGGACT	61	63	91	5

Supplementary Table S2. Primer set targeting antibiotic resistance genes.

Gene	Forward primer (5'-3')	Reverse primer (5'-3')	Size
AAC(6')-Ib	GCCCAGTCGTACGTTGC	CTTTGCCCAGTTGTGATGC	112
AAD	GATGAGCGAAATGTAGTG	GGCAGCGACATCCTTCG	84
ACC	CCGATTGTTCCCCCGTTA	GATTGAGTAGTTTTTGTTAGCCA	141
ACT	GGATGAGGTCACGGATA	CCAAAAAGACCGATGCT	111
aph2	TTTRTGCTTGCTGGATTGTA	TGCTTTTCTTTCTGCTACTTC	106
ccrA	CACAGAAAAGCGTAAAAATA	GCGAGGGATACATAAGT	79
cepA	AGACTTATACATTTATCCATTATC	TATTTCGGCTTTTTTCCC	117
CMY	TATGTACCAGGGATTAGGCTG	CTCAACGGCGGGAAGC	109
CTX-M-1	AGGAAGTGTGCCGCTGT	AATTATCTGCTGTGTTAATCA	113
CTX-M-2	TGCCGAAATCATGGGTAG	GGTTGGGTAAAGTAGGTCAC	124
CTX-M-25	CAGTAAAGTGATGGCGGTA	AATGGGGTTGTAGTTAATC	112
CTX-M-9	ACTACGGCACCACCAATG	TGCGGCTGGGTAAAATAG	85
DHA	TAAGCCCTATTATTTCAATTATG	CTCTTTTTTCGCCACAG	139
DIM	GTACATTGGATTCGTAAAAAT	CGTAGAAATTGATTGGTCA	111
ermB	ATCTATTCAACTTATCGTCAGA	TCCCAACAATTTTATACCTCT	114
ermF	AATTAGAACCTACACAAAAGTTAT	ACCTACCTCATAGACAAG	101
FOX	SAAAGGTTCCGCCTT	GCATCTTGTCATTCGAATC	107
GES	CACCTCGACCCACACCA	CCAACAACCCAATCTTT	93
GIM	AAGTTATAAAAATTGAAGATGGAGT	AAGGTGTGTCGATAATATAGGCT	129
IMI	GYAAATCATTTTCRTACARAGCA	GTGATGGGTGAATRGAAC	163
IMP	GGAATAGAGTGGCTTAATT	AACTTCAATTTTATTTTTAACTAG	144
KPC-2	TCTAGTTCTGCTGTCTTGTC	AGTCCTGTTCGAGTTTAG	101
MCR-1	CTCCAAAATGCCCTACAGA	CTTAACAAAAGCCACAAG	117
mecA	AACAAGTTCCAGATTACAAC	GCCAACCTTTACCATCG	121
mecC	GTTCCATACCATTAGTTAATATAT	GCTTTAGAAAATAACGGAAATAT	121
MOX	TGCACCACACCTATGTC	GCTTGTCCTCTTTCGA	75
NDM	CAGCACACTTCCTATCTCG	GGTCCAGGCGGTATCGA	102
OXA-1	GAAAACACCATAGAGAACA	TATAATAAACCCTTCAAACCA	126
OXA-10	TTTTCTGGTGTGGGAACTGAG	GATTTTGGTGGGAATGGATTTTC	150
OXA-23	TTCAAACAGATAAAAAAATTAATC	CGATCAGGGCATTCAACATT	102
OXA-24	ACTCAAGAAGAAGTTAAAAAAATG	CCAGTCAACCAACCTACCT	113
OXA-48	GTATCACAATAAGTTACACGT	CCAGTTTTAGCCCGAATAAT	102
OXA-58	TGCCTTTTAAACCTGAAGT	TTTTCAACAAAACCCACATACCA	142
PER	GAGTTTAGTGTTCCAGTGC	GTCTCCTTTATACCCATAGAC	140
qnrA	TATCAGTGTGACTTCAGCC	CCTTGAAACTGGCATCGC	127
qnrB	CGTTCAGTGGTTCAGATCT	AATTGGTCAGATCGCAATG	87
qnrS	GTGTGATTTAAAAGGKGCC	CCKCTCCATATTGGCATA	121
SHV	TGCAGTGGATGGTGGAC	CCAAGCAGGGCGACAAT	127
SPM-1	TTACAAGAAGATGGGCG	AACAGGATGGGAACTCAGAATC	145
TEM	CGCCGCATACACTATTCTC	GTCAGAAGTAAGTTGGC	143
tetQ	GTAGAAGATATGGAAGATTTTC	GACGGAGGATTTGAGAG	102
vanA	CGGAAAAAGGCTCWGAA	CGTTATCTTGTAAAAACATATC	150
vanB	GGTAAAATCCGCAATAGAAAT	CTTCCCATTCCGTACAT	122
VEB	TCAAGACCTTTTGCCTAAA	AAGAATTTTTGAACAGAATCAGT	168
VIM	GGTCTACCCGTCCAATGG	CCGCTGTGTTTTTCGCA	86

Supplementary Table S3. Single-stranded synthetic targets complementary to the G-quadruplex padlock probes.

Name	Target sequence (5'-3')
AAC(6')-Ib_tar	GGGACGGATGGTGGGACGTTGCTCTTGGAAGCG
ACC_tar	ATCTTCTTAGCAGGAACAAACGCCAGCATCACGATCCCC
ACT_tar	TAAAGACGCGTTGTGCCATGCTGGCGTTGGCG
aph2_tar	TGATAGTATTTTAATACCTTCCTGCCTTCCGGCGCCTTATGC
CMY_tar	TGCCAATGCCACTTTGCTCGGCGGGAAGCGC
CTX-M-1_tar	AACGGCGGACGTACAGGCCGCTGTATGCGCA
CTX-M-25_tar	GTGCTTAAGCAAAGTGAAACGGGCGGTAGCGGCG
CTX-M-9_tar	GCACGACCCTGCGGACCAGAACCAGCGGC
DHA_tar	ACCCAGCACACCTGTGATCGCCACAGAAACCGC
GES_tar	TGTCGCGTCTCCCGTTAGGAAAACCCGCTCGTAG
GIM_tar	AACCCATTCGAATCAACTAACCGGCTTGATTATTATCCAGAACTACC
IMI_tar	TCCATTATGTAGTTCTTTCAAAGGCTCGTACAAAGCAAATGAACGATT
IMP_tar	CCCCACGTATGCATCTGAAAGAGTGGCTTAATTCTCGATCTAT
mecA_tar	CAAAAAATATTAACAGCAATGATTGGGACAACTTCACCAGGTTCAACT
MOX_tar	ATGGCCTGCTTCGGCGCCATAGGCATAACTCGCC
OXA-1_tar	ATTTGCTGTGAATCCTGCACACCATCCGTTTTGTAAGGTTCT
OXA-10_tar	TGGTGGGTTGGGTGGGGTCAAATCCTGGTGTCGCA
OXA-23_tar	TCTGTATTTGCGCGGCTTAAATGTAGAGGCTGGCACATAT
OXA-24_tar	AACACCCATTCCCCATCCACCAACCTACCTGTGGAGT
PER_tar	ATACTCGGTCTCGCACACTGCAGCAACTGCTGCA
qnrB_tar	TTCGACTTTCGACTGGCGTCCGGCGGCGAGTT
qnrS_tar	AGCAAACTTTGCCAATCAAGTGGGGCCAACTTTTCCCG
SHV_tar	CCGCTCGCCAGCTCCCCGCGCGCACC
SPM-1_tar	AGACGTGGTCGAGCGAAAGAAGATGGGCGCGG
TEM_tar	TCATGGTTATGGCAGCACTTGGCCGCAGTGTTATCAC
vanA_tar	TCTACAGCCGAGCGCTTCCACACGGGCTAGACC
VEB_tar	AGTCCGATTAAAGAGGAATTCCCACCTTTTGCCTAAAACGTGG

Supplementary Table S4. Additional primers.

Name	Sequence (5'-3')
Padlock probe primer	GCCCGAGGTAAGTAGT
Padlock probe primer RC	ACTACTTACCTCGGGC
